# Reduced Mass and Diversity of the Colonic Microbiome in Patients with Multiple Sclerosis and Their Improvement with Ketogenic Diet

**DOI:** 10.3389/fmicb.2017.01141

**Published:** 2017-06-28

**Authors:** Alexander Swidsinski, Yvonne Dörffel, Vera Loening-Baucke, Christoph Gille, Önder Göktas, Anne Reißhauer, Jürgen Neuhaus, Karsten-Henrich Weylandt, Alexander Guschin, Markus Bock

**Affiliations:** ^1^Laboratory for Molecular Genetics, Polymicrobial Infections and Biofilms, Section of Gastroenterology and Hepatology, Department of Medicine, Charité Universitätsmedizin BerlinBerlin, Germany; ^2^Institute of Molecular Medicine, Sechenov First Moscow State Medical UniversityMoscow, Russia; ^3^Outpatient Clinic, Charité Universitätsmedizin BerlinBerlin, Germany; ^4^Faculty of Veterinary Medicine, Centre for Infectious Diseases, University of LeipzigLeipzig, Germany; ^5^Charité Universitätsmedizin Berlin, Campus Virchow, Gastroenterology BerlinBerlin, Germany; ^6^Laboratory of Molecular Diagnostics, Russian Research Center for Molecular Diagnostics and Therapy, Central Research InstituteMoscow, Russia; ^7^Experimental and Clinical Research Center, A Joint Cooperation between the Charité Medical Faculty and the Max-Delbrueck Center for Molecular MedicineBerlin, Germany

**Keywords:** FISH, colonic microbiota, multiple sclerosis, biofermentation, ketogenic diet

## Abstract

**Background:** Colonic microbiome is thought to be involved in auto-immune multiple sclerosis (MS). Interactions between diet and the colonic microbiome in MS are unknown.

**Methods:** We compared the composition of the colonic microbiota quantitatively in 25 MS patients and 14 healthy controls.Fluorescence in situ hybridization (FISH) with 162 ribosomal RNA derived bacterial FISH probes was used. Ten of the MS patients received a ketogenic diet for 6 months. Changes in concentrations of 35 numerically substantial bacterial groups were monitored at baseline and at 2, 12, and 23/24 weeks.

**Results:** No MS typical microbiome pattern was apparent.The total concentrations and diversity of substantial bacterial groups were reduced in MS patients (*P* < 0.001). Bacterial groups detected with EREC (mainly *Roseburia*), Bac303 (*Bacteroides*), and Fprau (*Faecalibacterium prausnitzii*) probes were diminished the most. The individual changes were multidirectional and inconsistent. The effects of a ketogenic diet were biphasic. In the short term, bacterial concentrations and diversity were further reduced. They started to recover at week 12 and exceeded significantly the baseline values after 23–24 weeks on the ketogenic diet.

**Conclusions:** Colonic biofermentative function is markedly impaired in MS patients.The ketogenic diet normalized concentrations of the colonic microbiome after 6 months.

## Introduction

There is a growing awareness of the significance of the human microbiome in health and disease. Microbial colonization of the skin and epithelial surfaces protects from pathogens. The biofermentation in the colon delivers energy from digestive leftovers and synthesizes a broad spectrum of vitamins and hormone-like substances, which regulate metabolism and neuronal activity (Galland, 2014). The enormous variety of the healthy microbiome conveys antigenic diversity to the host shaping its immunity and autoimmunity (Berer and Krishnamoorthy, 2014).

An ever growing number of studies demonstrates the involvement of the colonic microbiome in obesity, digestive, endocrine, inflammatory, and auto-immune disorders including multiple sclerosis (MS) (Berer and Krishnamoorthy, 2014; Galland, 2014; Glenn and Mowry, 2016).

Topics, methods and results of these studies are well presented and should not be repeated here. However, the previous studies of the colonic microbiome in MS were restricted to identification of microbial patterns associated with disease (Miyake et al., 2015; Jangi et al., 2016; Tremlett et al., 2016). We found no literature on the quantitative evaluation of the colonic microbiome in MS patients. Microbial concentrations, however, are an important feature, which directly measures their functional contribution to colonic fermentation. The aim of this study was to compare the concentrations of different microbial groups in MS patients and healthy controls and to follow up changes in the colonic microbiome taking place during ketogenic diet.

The option of ketogenic diet was important for the following reasons: as long as complex microbiomes cannot be reliably transferred, maintained and tested *in vitro*, all data raised *in vivo* must remain observational. Therefore, interventions simultaneously affecting the microbiome and disease are necessary to unravel possible causality.

Ketogenic diet influences brain function, inflammation, immunity and the colonic microbiome. It is increasingly applied in clinical studies (Piccio et al., 2008; Kim et al., 2012; Choi et al., 2016). Different to fasting or mono-diets, ketogenic diet can be maintained over months and is well tolerated. Diet prescriptions are often circumvented in real life. The compliance of the ketogenic diet can be reliably verified through measurement of ketone bodies in blood and urine and cannot be falsified.

## Materials and methods

### Patients/samples

Fourteen healthy volunteers from the Laboratories of Centre for Infectious Diseases, Faculty of Veterinary Medicine, University of Leipzig and 25 patients with relapsing-remitting multiple sclerosis cared for at the Charité hospital were investigated for the composition of their colonic microbiome using fluorescence *in situ* hybridization ribosomal RNA based FISH probes available in public resources (Loy et al., 2016).

The study was reviewed and approved by institutional review board: Ethikkomission Ethikausschuss 1 an Campus Charite Mitte EA1/130/07.

After the baseline investigation, MS patients received a ketogenic diet for 6 months. The ketogenic diet was designed (1) to achieve a modest ketosis (≥500 μmol/L ß-hydroxybutyrate) in the blood, self-measured after dinner twice a week (FreeStyle Precision, Abbott Diabetes Care Ltd.), (2) to achieve a modest ketosis (≥500 μmol/L acetoacetate) in the urine, self-measured after dinner once a week (Ketostix, Bayer Consumer Care AG), and (3) to maintain compliance. Patients received a booklet with meal suggestions over 28 balanced days and were encouraged to ingest fat. An average daily intake of < 50 g carbohydrates, >160 g fat, and < 100 g protein was recommended. Patients received detailed information about nutritional facts, glycemic load and learned how to handle carbohydrates by an experienced nutritional coach during group based workshops on 3 weekends.

Ten of the MS patients who were recruited for the ketogenic diet were randomly selected for accompanying microbiome investigations. Stools samples were collected at baseline, week 2, 12, and after 6 months (week 23–25). The evaluation of the clinical effects on MS was not performed because of the low number of patients.

None of the enclosed probands received antibiotics or probiotics in the last 6 month preceding the study.

### Fish

Colonic microbiota were investigated using FISH with ribosomal RNA derived probes. Hybridizations were performed on sections of Carnoy fixated, paraffin embedded and otherwise not manipulated stool cylinders (Swidsinski et al., 2010). Four micrometers thick sections were placed on SuperFrost plus slides.

A Nikon e600 fluorescence microscope was used. The images were photo-documented with a Nikon DXM 1200F color camera and software (Nikon, Tokyo, Japan).

Bacterial concentrations of homogeneous populations were enumerated visually in one of the 10 × 10 fields of the ocular raster corresponding to 10 × 10 μm of the section surface at magnification of 1,000. This number was assigned to a concentration of × 10^9^ bacteria/ml, which was most equivalent to the calculation formula, that we had used previously (Swidsinski et al., 2010).

In case of uneven distribution of bacteria over the microscopic field, the positive signals were enumerated in 10 fields of the ocular raster along the gradient of distribution and divided by 10.

Bacteria were quantified using group specific C3 probes. The FITC marked universal probe was used in each hybridization to evaluate the number of all bacteria, C5 marked probes with a different specificity to C3 probes were used to determine the spatial relation of different bacterial groups to each other.

Only signals that hybridized with a specific FISH probe and the universal FISH probe, but did not hybridize with specific FISH probes from unrelated bacterial groups, were enumerated (Swidsinski, 2006).

### FISH probes

One hundred sixty-two bacterial FISH probes available from public resources were applied for the comparative analysis of the colonic microbiome in healthy controls and MS patients (Tables 1A,B). The names of the FISH probes are listed according to abbreviations of the probeBase resource (http://probebase.csb.univie.ac.at/node/8) (Loy et al., 2016) and the details to FISH probe specificity and hybridization conditions are given. The Fprau probe is described in reference (Suau et al., 2001).

**Table 1:** Applied FISH-probes.

**Part A**
**Substantial groups**
**Essential (*N* = 3)**
Erec482 (*Eubacterium rectale, Clostridium coccoides* group)
Bac303 (most *Bacteroidaceae*)
Fprau (*Faecalibacterium prausnitzii*)
**Individual pioneer (*N* = 4)**
Bif153 Genus *Bifidobacterium*
Cdif198 *Clostridium difficile*
Clit135 *Clostridium lituseburense* group including *C. difficile*
Ebac1790 *Enterobacteriaceae*
**Individual substantial (*N* = 28)**
ACI623 *Acidaminococcaceae* sp. (not the Selenomonas species)
AKK406 *Akkermansia*
Ato291 *Atopobium* cluster
Bbif186 *B. bifidum*
Blon1004 *B. longum*
Bputre698 *Bacteroides putredinis*
*Burkho Burkholderia* spp.
Ceut705 *C. eutactus, Coprococcus* sp.
Chis150 *Clostridium histolyticum*
Cor653 *Coriobacterium* group
Cvir1414 *Clostridium viride* group
Ecyl387 *Eubacterium cylindroides*
Ehal1469 *Eubacterium hallii*
Eram997 *Eubacterium ramulus*
Lab158 *Lactobacillus* sp., *Enterococcus* sp.
Muc1437 *Akkermansia muciniphila*
Myc657 *Mycobacterium* subdivision (mycolic acid-containing *actinomycetes*)
Phasco741 *Phascolarctobacterium faecium*
Pnig657 *Prevotella nigrescens*
ProCo1264 *Ruminococcus productus*
Rbro730 *Clostridium sporosphaeroides, Ruminococcus bromii, Clostridium leptum*
Rfla729 *Ruminococcus albus*
SFB1 Segmented filamentous bacteria
SNA *Sphaerotilus natans*
Strc493 most *Streptococcus* spp.
SUBU1237 *Burkholderia* spp., *Sutterella* spp.
Urobe63a *Ruminococcus obeum*-like
Veil 223 *Veillonella*
Ver620 *Verrucomicrobium*
**Part B**
**Includes probes with extremely low occurrence and concentrations and probes with uncharacteristic signals:**
Accidental groups (*N* = 88)
To low in occurrence and concentrations for statistical analysis
MIB661 mouse intestinal bacteria
AER66 *Aeromonas* spp.
Alac1438 *Anaerococcus lactolyticus*
ARC1430 *Arcobacter*
Avag1280 *Anaerococcus vaginalis*
Bbrel198 *B. breve*
Bden82 *B. dentium*
BFV530 *Bacteroides forsythus*
Bif1278 *Bifidobacterium* spp.
Burcep *Burkholderia cepacia*
CAP365 *Capnoytophaga* sp.
Capno *Capnocytophaga sputigena*
Cj490 *Campylobacter jejuni*
CLOBU1022 *Clostridium butyricum*,
Cperf 191 *Clostridium perfringens*
Cra757 *Clostridium ramosum* assemblage
Csac67 *Clostridium saccharogumia*
CST440 Group 1 clones closely related to *Clostridium stercorarium*
DSV1292 some *Desulfovibrio* and *Bilophila wadsworthia*
DSV687 most *Desulfovibrionales*
E.bar1237 *Eubacterium barkeri*
E.bif462 *Eubacterium biforme*
E.con1122 *Eubacterium contortum*
E.cyl461 *Eubacterium cylindroides*
E.cyl466 *Eubacterium cylindroides*
E.dol183 *Eubacterium dolichum*
E.had579 *Eubacterium hadrum*
E.len194 *Eubacterium lentum*
E.lim1433 *Eubacterium limosum*
E.mon84 *Eubacterium moniliforme*
E.mul *Eubacterium multiforme*
E.sab *Eubacterium saburreum*
E.ven66 *Eubacterium ventriosum*
Enfl84 *Enterococcus faecalis*
Enfm 93 *Enterococcus faecium*
Fnec996 *Fusobacterium necrophorum*
Fnuc133 *Fusobacterium nucleatum*
GAN1237 *Helicobacter ganmani*
Haeinf *Haemophilus influenzae*
HEP642 *Helicobacter hepaticus*
Hpy-1 *Helicobacter pylori*
Hyo1210 *Brachyspira hyodysenteriae*
Lbuc668 *Leptotrichia buccalis*
Lis1255 Genera Li*s*teria, *Brochothrix*
Lis637 Genus *Listeria*,
Lpara *Lactobacillus casei*
Lzeae *Lactobacillus zeae*
Pae997 *Pseudomonas* spp.
Pamic1435 *Parvimonas micra*
Pana134 *Peptostreptococcus anaerobius*
Pden654 *Prevotella denticola*
Pilosi1405 *Brachyspira pilosicoli*
Pilosi209 *Brachyspira pilosicoli*
Pint649 *Prevotella intermedia*
Pint657 *Prevotella intermedia*
Pnasa1254 *Peptoniphilus asaccharolyticus*
Pnhar1466 *Peptoniphilus harei*
Pnivo731 *Peptoniphilus ivorii*
POGI *Porphyromonas gingivalis*
Ppu *Pseudomonas* spp.
Ppu56a *Pseudomonas putida*
Ppu646 *Pseudomonas* spp.
PRIN *Prevotella intermedia*
Saga *Streptococcus agalactiae*
SAL3 Genus *Salmonella*
Sau *Staphylococcus aureus*
Saur327 *Staphylococcus*
Saur72 *Staphylococcus aureus*
Ser1410 Genus *Brachyspira*
SGD229 Genus D*esulfotomaculum*
Sita649 *Candidatus Sphaeronema italicum*
Spn *Streptococcus pneumoniae*
Spy *Streptococcus pyogenes*
Staaur *Staphylococcus aureus*
Staph747 *Staphylococcus* spp.
STEBA1426 *Sterolibacterium* lineage
Stemal *Stenotrophomonas maltophilia*
Strpyo (Streppyo) *Streptococcus pyogenes*
Sval428 Some *Desulfobulbaceae*
TM7305 subdivision 1 of candidate division TM7
Trep-D3-4 32 PT1 *Treponema refringens*
Trep-D4-4 32 PT3 clone DDKL-20 *Treponema refringens*
Trep-HW 170 PT6 clone DDKL-4 *Treponema phagedenis*
Trep-T5-4 32 PT2 *Treponema refringens*
Urobe63b *Ruminococcus obeum*-like
VEPA *Veillonella parvula*
VIB572a Genus *Vibrio*
Y *Yersinia*
**FISH probes with signals which could not be definitively assigned to a specific group of bacteria (*N* = 31)**
Alf 1b (Alpha) *Alphaproteobacteria*, some *Deltaproteobacteria, Spirochaetes*
Arch 915 *Archaea*
B for *Bacteroides forsythus*
B(T)AFO *Tannerella forsythensis*
Bact for *Bacteroides forsythus*
Bang198 *B. angulatum*
Bfra602 most *Flavobacteria*, some *Bacteroidetes*
Bmy843 *Bacillus*
BORR4 Genus *Borrelia*
Bvulg1017 *Bacteroides vulgatus*
CF319a most *Flavobacteria*, some *Bacteroidetes*
CFB560 subgroup of *Bacteroidetes, CFB* division
Clept1240 *Clostridium leptum*
Efaec *Enterococcus faecalis, Enterococcus sulfuricus*
Enc131 *Enterococcus* spp and other
Ent *Enterobacteriaceae* except *Proteus* spp.
FUS664 most *Fusobacterium* sp
FUSO *Fusobacterium* sp.
MIB724 mouse intestinal bacteria
PBR2 *Bifidobacterium breve*
PseaerA *Pseudomonas aeruginosa*
PseaerB *Pseudomonas aeruginosa*
Rint623 *Roseburia cecicola, Roseburia intestinalis*
Rrec584 *Roseburia genus*
SRB385Db *Desulfobacterales*
ssp F suc *Fibrobacter succinogenes* subsp. *Succinogenes*
STA *Staphylococcus* spp.
Str(STR2) *Streptococcus* spp
TRE308 *Treponema* sp.
TrepGen 725 S-S-*Trep* Genus725 (202)
TW652 *Tropheryma whippelii*
**FISH probes which delivered identical results to other probes (*N* = 8)**
EC1531 *E. coli*
ECO1167 (ECO 45A) *Escherichia coli*
ENT183 *Enterobacteriaceae*
GAM42a *Gamma-proteobacteria*
HGC69a *Actinobacteria* (high G+C Gram-positive bacteria)
Bif164 *Bifidobacteriaceae, Bifidobacterium* spp.
EubII *Phylum Planctomycetes*
EubIII *Phylum Verrucomicrobia*

Probes in Table 1 are alphabetically ordered to subgroups according to abundancy and specificity as described in the result section.

We performed hybridizations with all probes but excluded from analysis 31 of these probes, because they showed multiple uncharacteristic signals, in form and distribution not resembling bacteria or cross-reacting with non-related bacterial groups and eight FISH probes that were identical to related probes for the same species.

To reduce the number of unnecessary investigations, while following the impact of the ketogenic diet on the colonic microbiome, only 35 bacterial groups which were found to have substantial occurrence (in at least 20% of individuals) and concentrations (>10^9^ in at least one of the stool samples of one individual) were applied.

### Statistical analysis

Differences between groups were evaluated using the two sided *t*-Student *U*-test. Data are presented as means ± *SD, P* <0.05 was considered statistically significant.

## Results

### Eligibility of the FISH probes for analysis of the stool microbiome

Three bacteria detected with EREC (mainly *Roseburia*), Bac303 (*Bacteroides*), and Fprau (*Faecalibacterium prausnitzii*) probes were always present in healthy human controls and MS patients and contributed to about half of the colonic microbiome in each subject. We called these groups **essential bacteria**.

All other investigated bacterial groups were individual, detectable only in a subset of patients. We called them **individual bacterial** groups.

Twenty-eight of the individual bacterial groups were found in at least 30% of the probands (mostly 50–70%) in concentrations of higher than 10^9^ bacteria/ml. They contributed substantially to the colonic microbial mass. We called them **individual substantial groups**.

Four of the **individual** bacterial groups including Bif (*Bifidobacteriacae*), Ebac (*Enterobacteriaceae*), Clit (*Clostridium lituseburense*), and Cdif (*Clostridium difficile*) are often found prevalent in newborns, after antibiotic treatment and convalescence patients and thus represent bacterial groups with pioneer function. We evaluated these groups separately.

Individual bacterial groups detected with 88 FISH probes (Table 1B) were observed in one, maximal two individuals in marginal concentrations of ≤0.1 × 10^9^ ml. We called these **individual marginal** bacterial groups. Because of the uneven distribution of such bacteria over the stool cylinder, their quantification was highly unreliable.

Thirty-one of investigated FISH probes showed multiple uncharacteristic signals, in form and distribution, cross-reacting with unrelated bacterial groups. The appearance of these signals was not visually different in MS and healthy controls. Because of the uncertainty of what exactly is measured, we did not perform the quantitative analysis of the results detected with these probes.

Eight FISH probes, showed identical results with related probes for the same species/bacterial group, were also not quantitatively evaluated.

### Microbiome in healthy controls and MS patients prior to diet intervention

The morphologic appearance of single bacteria detected with corresponding FISH probes was the same in MS patients and healthy controls. The distribution of bacteria over the stool cylinder surface was not noticeably different. None of the investigated bacterial groups, including groups with unspecific signals, demonstrated prevalence or absence in MS patients, which could be interpreted in terms of Koch's postulates.

As long as bacterial groups were compared pairwise, the differences between MS and healthy patients were discordant, gradual and moderate, reaching only in 9 groups statistical significance (Table 2). The concentrations of most investigated groups in MS were decreased. Six groups were slightly increased and included Cor653 (*Coriobacterium group*), Cvir1414 (*Clostridium viride* group) Ehal (*Eubacterium hallii*), Ecyl387 (*Eubacterium cylindroides)*, Lab158 (*Lactobacillus* sp., *Enterococcus* sp.), Rfla729 (*Ruminococcus albus)* bacterial groups. Seven substantial individual bacterial groups had similar high concentrations in MS and healthy controls.

**Table 2:** Colonic microbiome in healthy and MS patients prior to and during the ketogenic diet.

**Week**	**Healthy**	**MS**				***t*-test**
	**A**	**B**	**C**	**D**	**E**	
		**Initial**	**2**	**12**	**23/24**	
	**Mean ± *SD* concentrations**	**Mean ± *SD* concentrations**	**Mean ± *SD* concentrations**	**Mean ± *SD* concentrations**	**Mean ± *SD* concentrations**	
Diversity of microbiome as % of substantial bacterial groups (35) positive in each patient	75 ± 15	48 ± 19	35 ± 13	38 ± 6.9	51 ± 10.6	A/B,C,D,E *P* < 0.001; B/C *P* = 0.03–0.05; E/B *P* = 0.07; E/C *P* < 0.001; E/D *P* = 0.05
All bacterial groups x10^9^ bacteria/ml	**85.4** ± **25.6**	**65** ± **23.1**	**25.1** ± **17.2**	**36.4** ± **16.8**	**83** ± **25.8**	A/B,C,D *P* < 0.001; A/E *P* = 0.7; B/C,D *P* < 0.001; E/B *P* = 0.02; E/C,D *P* < 0.0001
**ESSENTIAL**						
All Essential bacteria (*N* = 3)	**36.2** ± **14.7**	**24.6** ± **9.5**	**10.7** ± **8.4**	**16.1** ± **7.4**	**34.4** ± **10**	A/B,C,D *P* < 0.001; A/E *P* = 0.4; B/C *P* = 0.001; B/D *P* = 0.7; E/B *P* = 0.05; E/C,D *P* < 0.001
Erec482 (*Eubacterium rectale, Clostridium coccoides* group)	11.7 ± 6.9	6.7 ± 5.8	6.1 ± 2.8	6.4 ± 5.3	10.7 ± 7.3	A/B,C,D *P* = 0.02–0.002; A/E *P* = 0.6; B/C *P* = 0.2; B/D *P* = 0.9; E/B,C,D *P* = 0.03–0.02
Bac303 (*Bacteroides*)	12.9 ± 5.3	9.1 ± 4.9	3.4 ± 3.1	4.2 ± 2.7	11.4 ± 6.2	A/B,C,D P < 0.001–0.006; A/E P = 0.3; B/C,D P = 0.001-0.002; E/B P = 0.09; E/C,D P < 0.001
Fprau (*Faecalibacterium prausnitzii*)	11.6 ± 5.9	8.8 ± 5.1	2.7 ± 4.2	5.7 ± 4	12.2 ± 7.9	A/B *P* < 0.05; A/E *P* = 0.8; B/C,D *P* = 0.001; B/D *P* = 0.08; E/B *P* = 0.05; E/C,D *P* < 0.001–0.007
**INDIVIDUAL PIONEER**						
All Individual pioneer bacteria (*N* = 4)	**7.8** ± **5**	**6.7** ± **5.3**	**2.5** ± **3.2**	**1.9** ± **3**	**2.7** ± **4.3**	A/B *P* = 0.4: A/C,D *P* = 0.001–0.005; A/E *P* = 0.004; B/C *P* = 0.03; B/D *P* = 0.008; E/B *P* = 0.03; E/C,D P = 0.5-0.2
Ebac1790 (*Enterobacteriaceae*)	0.25 ± 0.8	0.5 ± 1.4	0.4 ± 1.2	0.05 ± 0.2	0.15 ± 0.8	All *P* > 0.25
Bif153 (Genus *Bifidobacterium*)	7.1 ± 5.5	5.7 ± 4.9	2.1 ± 2..4	1.8 ± 3	2.1 ± 3	A/B *P* = 0.3; E/C,D *P* > 0.85; A/C,D *P* < 0.001; B/C,D *P* = 0.02–0.03; B/E *P* = 0.008; E/A *P* < 0.001
Clit135 (*Clostridium lituseburense* group including *C. difficile*)	0.5 ± 0.86	0.4 ± 1.2	0	0.03 ± 0.09	0.2 ± 0.6	All *P* > 0.1
Cdif198 (*Clostridium difficile*)	0.04 ± 0.09	0.04 ± 0.2	0	0.001 ± 0.003	0.2 ± 0.6	All *P* > 0.3
**INDIVIDUAL SUBSTANTIAL**						
All Individual substantial bacteria (*N* = 28)	**41.7** ± **17.3**	**33.8** ± **16.8**	**11.8** ± **9**	**18.3** ± **11.6**	**46.9** ± **18.9**	A/B *P* = 0.08; A/C,D *P* < 0.001; A/E *P* = 0 × .3: B/C *P* < 0.001; B/D *P* = 0.006; E/B *P* = 0.02; E/C,D *P* < 0.001
ACI623 (*Acidaminococcaceae* sp. not the Selenomonas species)	1.4 ± 1.9	0.6 ± 1.4	0.4 ± 1.3	0.2 ± 0.4	0.5 ± 0.7	A/B, *P* = 0.0.1; A/C *P* = 0.2; A/D *P* = 0.08; A/E *P* = 0.01; B/C *P* = 0.8; B/D *P* = 0.4; E/B *P* = 0.7; E/C,D *P* = 0.5–0.7
AKK406 (*Akkermansia*)	2.3 ± 3.7	1.1 ± 2.7	2.1 ± 3.8	0.7 ± 2.2	1 ± 1.7	A/B *P* = 0.2; A/C *P* = 0.7; A/D *P* = 0.3; A/E *P* = 0.2; B/C *P* = 0.2; B/D *P* = 0.9; E/B *P* = 0.7; E/C,D *P* = 0.4–0.9
Ato291 (*Atopobium* cluster)	3.8 ± 2.9	2.9 ± 3.1	0.45 ± 0.5	2.6 ± 2.3	4.1 ± 3.1	A/B *P* = 0.3; A/E *P* = 0.7; B/C *P* = 0.02; E/B *P* = 0.2
Bbif186 (*B. bifidum*)	0.3 ± 0.9	0.1 ± 0.4	0	0	0.05 ± 0.2	A/B *P* = 0.3; A/E *P* = 0.2; B/E *P* = 0.6
Blon1004 (*B. longum*)	0.7 ± 1.2	0.3 ± 0.8	0.2 ± 0.4	0	0.5 ± 0.9	A/B *P* = 0.3; A/E *P* = 0.7; B/C *P* = 0.7; E/B *P* = 0.5
Bputre698 (*Bacteroides putredinis*)	0.8 ± 1.6	0.06 ± 0.2	0.05 ± 0.2	0	0.3 ± 0.8	A/B *P* = 0.03; A/E *P* = 0.2; B/C *P* = 0.9; E/B *P* = 0.2; E/C *P* = 0.4
Burkho (*Burkholderia* spp.)	0.7 ± 0.7	0.7 ± 1.2	0.8 ± 0.7	0.001 ± 0.03	0.9 ± 1.5	A/B *P* = 0.98; B/C *P* = 0.5; E/B *P* = 0.7; E/C *P* = 0.4
Ceut705 (*C. eutactus, Coprococcus* sp.)	3.0 ± 4.4	1.7 ± 2.9	0.15 ± 0.3	0.2 ± 0.4	1 ± 2	A/B *P* = 0.2; A/C *P* = 0.05; A/E *P* = 0.07; B/C *P* = 0.1; E/B *P* = 0.4; E/C,D *P* = 0.2
Chis150 (*Clostridium histolyticum*)	0.6 ± 1.2	0.05 ± 0.2	0.03 ± 0.1	0.04 ± 0.1	1 ± 2.3	A/B *P* = 0.03; A/E *P* = 0.5; B/C,D *P* = 0.8–0.9; E/B *P* = 0.08
Cor653 (*Coriobacterium group*)	0.5 ± 0.8	0.8 ± 1.2	0.5 ± 1.1	0.1 ± 0.3	0.09 ± 0.2	A/B *P* = 0.0.3; A/C, *P* = 0.9; A/E *P* = 0.04; B/C *P* = 0.5; E/B *P* = 0.02; E/C,D *P* = 0.1
Cvir1414 (*Clostridium viride group*)	1.9 ± 2.1	2.2 ± 2.7	0.5 ± 0.4	2 ± 2.6	4.7 ± 3.6	A/B *P* = 0.7; A/C *P* = 0.04; A/E *P* = 0.002; B/C *P* = 0.05; E/B,C,D < 0.01
Ecyl387 (*Eubacterium cylindroides*)	0.7 ± 0.5	1 ± 1.2	0.3 ± 0.7	0.3 ± 0.6	0.3 ± 0.8	A/B *P* = 0.0.1; A/E *P* = 0.1; B/C *P* = 0.1; E/B *P* = 0.05
Ehal1469 (*Eubacterium hallii*)	0.6 ± 0.9	1.4 ± 1.9	1.2 ± 1.2	1.7 ± 2.1	2.9 ± 2.1	A/B *P* = 0.04; A/E *P* < 0.001; B/C *P* = 0.7; E/B,C *P* < 0.01
Eram997 (*Eubacterium ramulus*)	0.3 ± 1.3	0.09 ± 0.2	0	0	0.2 ± 0.8	A/B *P* = 0.0.4; A/E *P* = 0.8; B/C *P* = 0.2; E/B *P* = 0.5
Lab158 (*Lactobacillus* sp., *Enterococcus* sp.)	0.1 ± 0.2	1.2 ± 2.9	0.1 ± 0.3	0.02 ± 0.02	0.1 ± 0.4	A/B *P* = 0.05; A/E *P* = 0.7; B/C *P* = 0.3; E/C,D *P* = 0.1
Muc1437 (*Akkermansia muciniphila*)	2.8 ± 3.9	1.7 ± 3.4	1.8 ± 4	0.5 ± 1.5	1 ± 1.2	A/B *P* = 0.3; A/E *P* = 0.1; B/C *P* = 0.9; E/B *P* = 0.5
Myc657 (*Mycobacterium* subdivision, mycolic acid-containing *Actinomycetes*)	3.1 ± 1.5	3 ± 3.1	1.2 ± 1.6	3.3 ± 3	4.2 ± 3.8	A/B *P* = 0.9; A/E *P* = 0.2; B/C *P* = 0.09; E/B *P* = 0.3
Phasco741 (*Phascolarctobacterium faecium*)	0.6 ± 0.9	0.6 ± 2.1	0	0.4 ± 0.8	0.4 ± 0.7	A/B *P* = 0.9; A/E *P* = 0.7; B/C *P* = 0.4; E/B *P* = 0.7
Pnig657 (*Prevotella nigrescens*)	2.2 ± 3.7	0.2 ± 0.6	0	0	1.1 ± 3	A/B *P* = 0.01; A/E *P* = 0.4; B/C *P* = 0.3; E/B *P* = 0.3
ProCo1264 (*Ruminococcus productus*)	0.7 ± 2	0.02 ± 0.08	0	0.09 ± 0.3	0.8 ± 1.8	A/B *P* = 0.08; A/E *P* = 0.7; B/C *P* = 0.5; E/B *P* = 0.03
Rfla729 (*Ruminococcus albus*)	2.2 ± 3.2	5.1 ± 3.2	1.4 ± 2.3	0.6 ± 1.3	6.5 ± 4.4	A/B *P* = 0.002; A/E *P* < 0.001; B/C *P* = 0.02; E/B *P* = 0.2
SFB1 (*Segmented filamentous bacteria*)	2.3 ± 3.3	1.5 ± 3.8	0.4 ± 0.8	1.9 ± 3.5	3.7 ± 4.1	A/B *P* = 0.4; A/E *P* = 0.2; B/C *P* = 0.4; E/B *P* = 0.1
SNA (*Sphaerotilus natans*)	4.3 ± 3.7	3.2 ± 3.7	0.3 ± 0.4	0.001 ± 0.003	5.7 ± 4.8	A/B *P* = 0.3; A/E *P* = 0.2; B/C *P* = 0.02; E/B *P* = 0.05
Strc493 (most *Streptococcus* spp.)	1.3 ± 3.3	0.8 ± 1.5	0.01 ± 0.03	0.4 ± 1.2	1 ± 1.7	A/B *P* = 0.4; A/E *P* = 0.8; B/C *P* = 0.1; E/B *P* = 0.6
SUBU1237 (*Burkholderia* spp., *Sutterella* spp.)	1.7 ± 2.6	0.6 ± 1.2	0.1 ± 0.3	0.1 ± 0.3	1.4 ± 1.6	A/B *P* = 0.08; A/E *P* = 0.7; B/C *P* = 0.2; E/B *P* = 0.1
Urobe63a (*Ruminococcus obeum*-like)	1.6 ± 2.3	1.6 ± 2.8	0.06 ± 0.2	0.7 ± 1.8	2.1 ± 1.9	A/B *P* = 0.9; A/E *P* = 0.5; B/C *P* = 0.1; E/B *P* = 0.5
Veil223 (*Veillonella*)	0.1 ± 0.3	0.04 ± 0.2	0	0.2 ± 0.4	0.16 ± 0.3	A/B *P* = 0.2; A/E *P* = 0.8; B/C *P* = 0.5; E/B *P* = 0.2
Ver620 (*Verrucomicrobium*)	1.7 ± 3.9	1 ± 2.7	0	1.9 ± 3.6	1.2 ± 2.4	A/B *P* = 0.5; A/E *P* = 0.6; B/C *P* = 0.2; E/B *P* = 0.9

Due to uncertain occurrence and low concentrations, which was difficult to quantify, we did not compare the single marginal bacterial groups in MS patients and controls quantitatively. No rise of single marginal groups in MS patients was observed.

The difference in the microbiome of MS patients and healthy controls became striking, when concentrations of numerically substantial groups were summarized and considered as a whole (Table 2, marked in bold). The diversity of all substantial groups in MS patients was reduced by 36% (*P* < 0.001), the mean sum concentrations of all substantial bacterial groups were reduced by 24% (65 vs. 85.4 × 10^9^/bacteria/ml., *P* < 0.001) compared to healthy controls. The decrease in concentrations was most profound in the essential bacteria group (32%) and less impressive in individual substantial (19%) and pioneer groups (14%).

### Changes of the colonic microbiome with the ketogenic diet in 10 MS patients

The changes in the microbiome during the ketogenic diet were univocal. Except for *Akkermansia*, all groups of bacteria demonstrated consistently more or less marked decreases, leading to a reduction of the total bacterial concentrations of the substantial bacteria from 65 to 25 × 10^9^ bacteria per ml at week 2. Some of the bacterial groups fell below detection level, resulting in further decline of the bacterial diversity from 48 to 36%.

However these tendencies were temporary.

The total bacterial concentrations in MS patients started to increase at week 12, reaching values typical for healthy controls at week 23/24, being then significantly higher than bacterial concentrations in MS patients prior to diet and statistically not different to mean bacterial concentrations in healthy controls (*P* = 0.7). This increase was consistent for all but the pioneer bacterial groups and *Akkermansia*. The concentrations of pioneer bacterial groups fell and remained low, the concentrations of *Akkermansia* increased initially but then declined during the ketogenic diet.

## Discussion

Previous investigation, using high throughput DNA sequencing technologies in large scale 16S rRNA or shotgun metagenomic sequencing, demonstrated miscellaneous changes in the composition of the colonic microbiome, which correlated with MS, MS-onset, -therapy or -relapse (Kim et al., 2012; Berer and Krishnamoorthy, 2014; Galland, 2014; Miyake et al., 2015; Glenn and Mowry, 2016; Jangi et al., 2016; Tremlett et al., 2016). Both single bacterial groups were found differently represented and also the whole microbiome was aberrantly composed. While the alpha diversity of MS and the healthy microbiome was similar, the beta diversity differed significantly. In ecology, alpha diversity expresses the mean species diversity in sites or habitat at a local scale, while beta diversity is the ratio between regional and local diversity. The differences indicated shifts in composition of the MS microbiome. The meaning of these observations is unclear. The pure occurrence of bacteria does not automatically mean that they are relevant or biochemically active. The vacant niches may be occupied by chance.

Although we applied publically available FISH probes as broadly as possible and included all groups covering numerically substantial components of the colonic microbiome, no conclusions to the entire biodiversity are possible, since FISH reliably detects only bacteria in concentrations of higher than 10^5^ per ml.

However, while sequence analysis is perfect for identification of specific occurrence patterns, its information on physical abundance and contribution of bacteria to biofermentation is poor. Abundance of bacteria within the fecal mass however directly expresses their biofermenting power.

Our data quantifying microbial participants clearly demonstrate the impaired colonic function in patients with MS. Both concentrations and biodiversity of numerically substantial bacterial groups were markedly reduced in MS. Essential bacteria were most, individual substantial bacteria less depleted, while pioneer bacteria were nearly unchanged.

This grading of suppression matches well with the proposed role of these groups for colonic function. Essential bacteria are present in every healthy person in large concentrations, contributing roughly to approximately half of the mass of the colonic microbiome. They are obviously important for colonic fermentation and represent main fermentative groups in human. The individual substantial bacterial groups are present only in subsets of healthy persons in varying concentrations, which are each distinctly lower than those of the essential bacterial groups. Their presence is dispensable for colonic fermentation. However, their diversity is high, composition specific for each subject, indicating that they fulfill special tasks not covered by the essential bacterial groups. Despite markedly lower concentrations, when compared to essential bacterial groups, the individual substantial groups constitute together another half of the colonic biomass.

Pioneer bacteria are usually found in low concentration in healthy adults. Their concentrations are high in newborns after antibiotic treatment and in convalescence, while the colonic microbiome is reshaped (Swidsinski et al., 2016).

The fall in concentration in MS patients was gradual, decreasing from essential to individual substantial and further to pioneer bacterial groups. This suggests that the suppression is not due to the loss of responsible microbial groups or reshaping of the microbiome. It presumably results from the general downregulation of the colonic biofermentative function and affects mainly biofermentative active groups, leaving bacterial groups with other specific tasks untouched.

We observed no changes in the microbiome that could be specific for MS.

While mean concentrations of all essential bacteria detected with EREC (mainly *Roseburia*), Bac303 (*Bacteroides*), Fprau (*F. prausnitzii*) probes were consistently reduced in MS, the shifts in individual substantial bacterial groups were multidirectional with concentrations of some bacterial groups unchanged, increased or reduced when compared to healthy controls. Although some of the differences reached the level of statistical significance, the fluctuations in concentrations of individual substantial bacterial groups were moderate and in the range of those, when unmatched groups of subjects are compared with each other. Similar fluctuations are documented while describing microbiome composition in other pathologies such as diarrhea, irritable bowel syndrome, inflammatory bowel disease, and others (Mai et al., 2016; Swidsinski et al., 2016).

Since the microbiome is influenced by a multiplicity of racial, occupational, social, regional, and geographic factors, matching for all of them would be an impossible task. Mean values raised in small cohorts should therefore be critically evaluated, even when they occasionally prove to be statistically highly significant (Mai et al., 2016). Our data allow definitively no conclusions to whether the observed alterations in concentrations of colonic bacteria precede, result, specifically accompany MS or rely on independent coincident processes such as aberrant immunity, impaired digestion or simply changed behavior. However, they clearly demonstrate that the perturbation of the microbiome in MS is not inherent and inevitable but can be definitively corrected with diet, supplementation and other means as assumed previously (Tanca et al., 2015).

After 6 months on the ketogenic diet, the sum concentrations of the substantial microbial groups in MS patients increased significantly when compared to the period prior to intervention (83 vs. 65 × 10^9^ bacteria/ml; *P* = 0.02) and became indistinguishable from the healthy group (83 vs. 85.4 × 10^9^ bacteria/ml. *P* = 0.7).

Our data also demonstrate that the tracking of the changes in the microbiome should not be restricted to a single time point, but needs surveillance over longer periods of time.

The reduction of microbial concentrations observed 2 weeks after the start of the diet was dramatic, the ranges of suppression were comparable to antibiotics effects (Swidsinski et al., 2016). Concentrations of some individual substantial groups fell under detection limit, leading to further drop in the microbial diversity from mean 48 to 35 percent. However, the processes behind dietetic and antibiotic effects are principally different.

The improvement with antibiotic treatment occurred only after the end of treatment, while with the ketogenic diet, the improvement occurred in succession of the diet and the long-term effects of diet were opposite to the immediate response to the intervention. Although the diversity of the microbiome did not reach the values typical for the healthy population, at the end of the observation period, after 6 months on the ketogenic diet, it completely recovered as compared to basic values prior to treatment.

Obviously the increase in microbial concentrations was mainly due to improvement of colonic function and achieved by preexisting microbial groups. Typical for convalescence after depletion of the colonic microbiome following antibiotic use, stroke or inflammation is a temporary excess of pioneer bacterial groups. Such excess was not observed with the ketogenic diet. In contrast, the pioneer bacterial groups remained reduced over the whole duration of the ketogenic diet (*p* < 0.05–0.008), indicating an absence of substantial microbiome reshaping.

Although the concentrations and the biodiversity of colonic microbiota are strong markers of the intensity of the microbial metabolism, the shifts in bacterial groups *per se* do not reveal the exact metabolic changes taking place. The role of single substances and metabolites in neurologic disorders is still to be unraveled and follow our preliminary observations.

Summarizing, we state that colonic microbiome and neuropathology are closely interrelated. Concentrations of numerically substantial biofermentative bacteria are significantly reduced in MS patients. The microbial shifts can be reliably quantified and monitored by FISH under ambulatory conditions. The ketogenic diet for 6 months completely restored the microbial biofermentation mass and is an interesting interventional tool for prospective clinical studies.

## Author contributions

AS, MB,YD, and AG designed the study, YD, JN, AR, KW, and ÖG conducted the study, ÖG and VL critically revised the manuscript, and AS, AG, CG, and AR. performed FISH, CG, JN statistically analyzed the data. All authors contributed to conception of the work, revising of the data, shaping of the manuscript and approved the final draft submitted.

### Conflict of interest statement

The authors declare that the research was conducted in the absence of any commercial or financial relationships that could be construed as a potential conflict of interest.
